# Glycosylated Hemoglobin as an Independent Prognostic Marker in COVID-19 Patients With Diabetes Mellitus

**DOI:** 10.7759/cureus.28634

**Published:** 2022-08-31

**Authors:** Gopakumar Dalia, Alagesan Chellappillai Vaiera Manigandan, Seetharaman Ranganathan Rangabashyam

**Affiliations:** 1 General Medicine, Vinayaga Mission Kirupananda Varriyar Medical College and Hospital, Salem, IND

**Keywords:** hyperosmolar hyperglycemic state, diabetic keto acidosis, covid 19, diabetes mellitus, glycosylated hemoglobin

## Abstract

Objective

The main objective of this study is to analyze the severity of coronavirus disease 2019 (COVID-19) among patients with diabetes mellitus using glycosylated hemoglobin (HbA1c) as a prognostic marker in predicting the outcome among these individuals.

Methods

This was a retrospective, observational study conducted in a tertiary care center during the first and second waves of COVID-19 in Salem, Tamil Nadu, for a period of one year (September 2020-September 2021). Numerous variables, including socio-demographic data, vitals, laboratory and radiological investigations, and end variables like mortality and morbidity due to COVID-19, were studied.

Results

Out of the 754 people admitted, 253 were diabetic, among which only 65 individuals fulfilled the criteria for participation. Among the 65 patients in the study, 21 had mild disease, 28 had moderate disease, among whom, two patients had HbA1c less than 7, 10 had between 7 and 8, 11 had between 8 and 10, and five had above 10, and 16 had severe disease, among whom one patient had HbA1c less than 7, 4 between 7 and 8, seven between 8 and 10, and four above 10. This was statistically significant (0.005).

Conclusion

The incidence of mortality was high among patients with prolonged uncontrolled diabetes mellitus with high HbA1c and among patients presenting diabetic complications like diabetic ketoacidosis (DKA), euglycemic ketoacidosis, and hyperglycemic hyperosmolar syndrome (HHS).

## Introduction

The coronavirus disease 2019 (COVID-19) pandemic has brought incalculable damage to the global economy and health care. Even the developed countries' health care systems became overwhelmed by the disease burden. COVID-19 predominantly affected the elderly population [1]. Glycosylated hemoglobin gives a better idea of the patient’s glycemic control [2,3]. In this study, we tried to use glycosylated hemoglobin levels as an independent indirect marker in predicting the outcome in COVID-19 patients [1-3]. Patients with co-morbidities, such as hypertension, diabetes, coronary artery disease, and chronic kidney disease, had higher mortality and morbidity rate compared to normal individuals [2-6]. Uncontrolled diabetes mellitus was one of the major risk factors affecting the outcome in patients during the COVID-19 pandemic [3-5].

Hyperglycemia in COVID-19 patients had severe implications like increased duration of ICU stay, increased need for mechanical ventilation, and a substantial rise in inflammatory markers [3-5]. Patients with diabetes or hyperglycemia had mortality and severity rates two to four times greater than COVID-19 patients without diabetes [2-6]. In COVID-19 patients with diabetes, compromised immune response to viral infection was the primary cause of mortality [2-7]. Elevated levels of blood sugar led to decreased intracellular degradation of bacteria by neutrophil chemotaxis and phagocytosis, leading to increased viral binding affinity and facilitating entry while decreasing viral clearance [3-8]. To minimize medical complications and mortality of the treatment in COVID-19 patients with diabetes, an integrated team approach is required [7-9].

## Materials and methods

This study was an observational analytical study done in a tertiary care hospital in Salem, Tamil Nadu, for a period of one year (September 2020 to September 2021). All the patients who were admitted to the COVID-19 ward during the study period through reverse transcription-polymerase chain reaction (RT-PCR) or chest CT findings and had diabetes mellitus or presented diabetic ketoacidosis, euglycemic ketoacidosis, hyperosmolar hyperglycemic syndrome, or hyperglycemia (capillary blood glucose >250 mg/dl) to the emergency department were included in the study. Patients who were not willing to participate in the study, patients with end-organ damage or established systemic hypertension, chronic kidney disease, acute kidney injury, hypothyroid and hyperthyroid, coronary artery disease, cerebrovascular accident, or chronic liver disease, and patients who received steroid or partial treatment outside the hospital during admission was excluded from the study.

Sampling technique

Out of the 754 COVID patients admitted to our hospital in India, 253 had diabetes, out of which 175 patients were excluded for various reasons, 86 patients had received partial treatment or steroid therapy, 34 patients had presented acute kidney injury (AKI), 55 patients had other comorbid conditions, and only78 patients who fulfilled the study criteria were taken. Out of the 78 individuals, only 65 participated in the study. The eligible study participants were included by a convenient sampling technique. With the assumption of COVID-19 prevalence as 50% and absolute error as 5%, the calculated sample size was 65.

Data collection

Data were collected by interviewing the patients using a semi-structured questionnaire and visualizing and cross-checking the hospital records. The study questionnaire contains numerous variables, including socio-demographic characteristics, such as name, age, religion, gender, vitals, previous and recent treatment history, comorbid conditions, investigations (glycosylated hemoglobin (HbA1c), D-dimer, C-reactive protein (CRP), erythrocyte sedimentation rate (ESR)), CT severity scores, and real-time reverse transcription-polymerase chain reaction (RT-PCR) test results. The outcome variable was morbidities like the need for oxygen therapy, duration of ICU and hospital stay, and death due to COVID-19.

Statistical analysis

The collected data were entered in Microsoft Excel (Microsoft Corporation, Redmond, WA) and the results were analyzed using SPSS (Statistical Package for the Social Sciences) version 23 (IBM Corp., Armonk, NY). Continuous variables were represented as mean with standard deviation, and frequency variables were represented as percentages. To find the test of significance between the frequency variables, the chi-square test was used. A p-value of less than 0.05 was considered statistically significant with a 95% confidence level.

## Results

Incidence

Age and Sex Distribution

Out of the 65 patients, 9.23% (6) patients had type 1 diabetes, and 90.76% (59) had type 2 diabetes. In this study, the age group ranged from 21 years to 65 years, as shown in Table 1. The mean age of the study population was 52.64. The SD was 52.64±14.10. The confidence interval (95%) was 52.64±3.428.

**Table 1:** Age and Sex Distribution DM: diabetes mellitus

Age (Years)	Male	Female
21–30	4	0
31–40	2	6
41–50	0	35
51–60	0	15
>60	0	3
Sex Distribution	TYPE I DM	TYPE II DM
Male	4	36
Female	2	23

The statistically significant age group in our study was between 41 to 60 years. Incidence was greater in middle-aged adults. This study was done mainly among the Indian population. This study population consisted predominantly of men around 40 and women around 25. In our study, we found statistical significance among both men and women.

**Figure 1:**
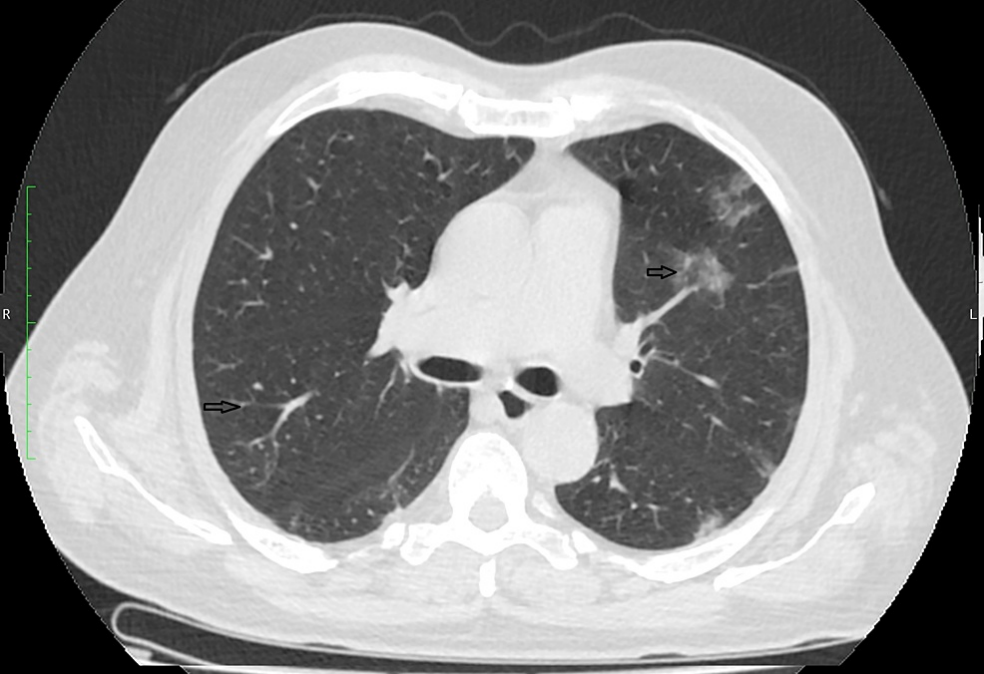
CT Chest Showing Small, Localized Ground-Glass Opacities in the Lung Peripheries

**Figure 2:**
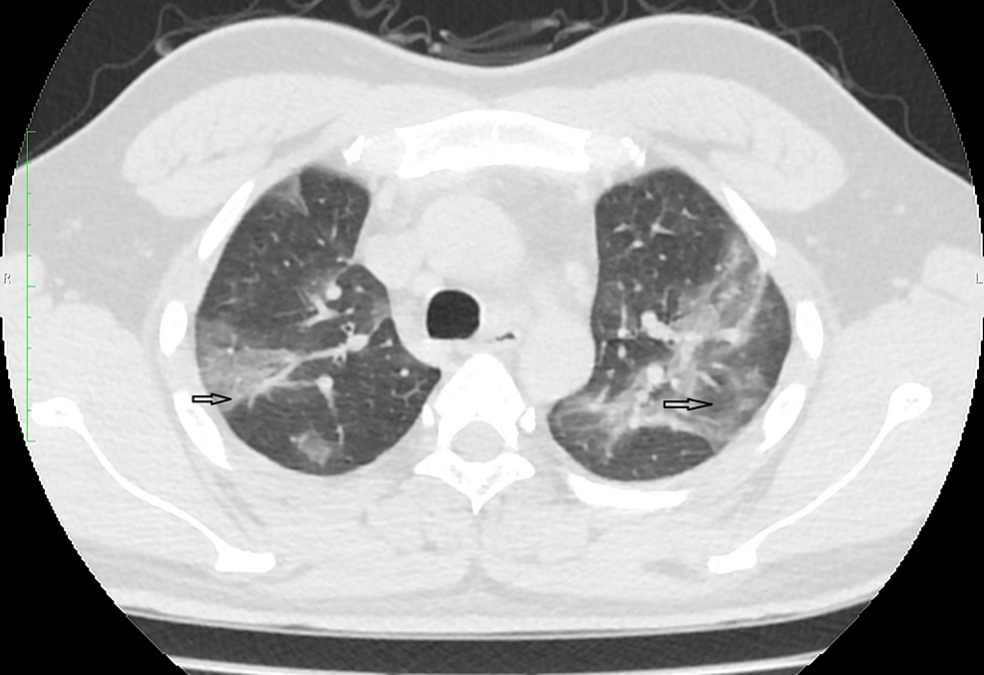
CT Chest Showing Diffuse Crazy Paving Pattern, Consolidation, and Septal Thickening

**Figure 3:**
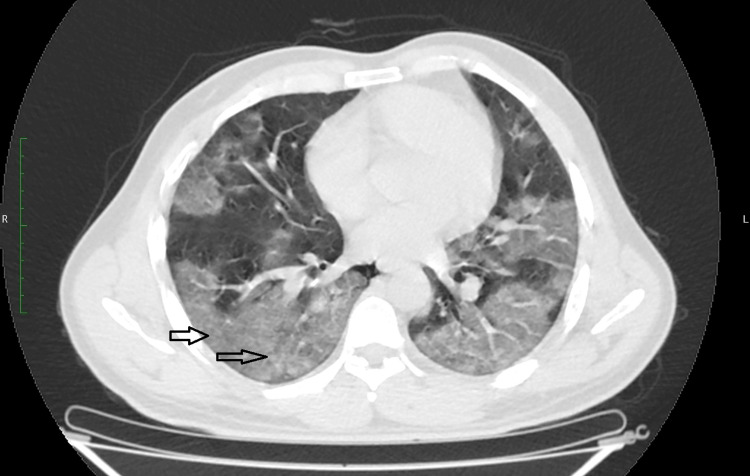
CT Chest Showing Heavy Opaque Consolidation and Marked Septal Thickening

In our study, we classified the patients into mild, moderate, and severe based on their disease severity (i.e., temperature, respiratory rate, room air saturation, and CT severity score). Patients in the mild category had a respiratory rate of 18-22 per min with saturation above 95% on room air and temperature <100.4℉, small localized ground-glass opacities in the lung peripheries as shown in Figure 1, and impaired six-minute walk test. Patients belonging to the moderate category had a respiratory rate of 22-30 per min with saturation above 90% but less than 95% on room air and temperature <104℉, and CT chest showing diffuse crazy paving pattern, consolidation, and septal thickening as shown in Figure 2. In the severe category, patients had a respiratory rate >30 per min with saturation <90% on room air with marked respiratory failure and high temperature >104℉, and CT chest showing heavy opaque consolidation and marked septal thickening as shown in Figure 3.

In our study, the mean age of patients in the mild severity group was 51.76, the mean age of the moderate group was 52.61, and that of the severe group was 53.55; this was found to be statistically significant, as shown in Table 2. The mean pulse rate per minute was 94.5 in mild, 102.43 in moderate, and 105.78 in severe cases.

**Table 2:** Disease Severity and Average of Various Parameters Across Each Group

PARAMETERS	CT SCORE						
	MILD		MODERATE		SEVERE		P-VALUE
	MEAN	SD	MEAN	SD	MEAN	SD	
AGE	51.76	15.10	52.61	14.67	53.55	12.54	0.05
PULSE RATE	94.5	11.34	102.43	17.8	105.78	16.65	0.062
SPO2	95.34	4.65	90.56	5.18	86.25	10.54	<0.0001
D DIMER	419.65	254.46	669.89	456.36	889.26	887.56	<0.0001
CRP	20.87	15.34	29.45	30.7	54.61	35.98	<0.0001
HbA1c	8.45	2.12	8.87	1.98	8.76	1.86	0.0056
HOSPITAL STAY	7.85	3.21	10.45	4.65	13.21	5.67	<0.001
ICU STAY	6.12	4.36	8.69	3.56	11.56	6.79	<0.001

The mean saturation in the mild group was 95.34, 90.56 in the moderate, and 86.25 in the severe group. The mean D-dimer in the mild group was 419.65, in the moderate group 669.89, and in the severe group 889.26; this was found to be statistically significant. The mean CRP level in the mild group was 20.87, in the moderate group 29.45, and in the severe group 54.61; this was found to be statistically significant. The mean HbA1c in the mild group was 8.45, in the moderate group 8.87, and in the severe group 8.76; this was found to be statistically significant.

HbA1c and disease severity

In our study, 16.9% (11) patients had HbA1c >10, 24.61% (16) had an HbA1c value between 8 and 10, 29.23% (19) between 7 and 8, and 29.23% (19) <7, as shown in Table 3.

**Table 3:** HbA1c and Disease Severity HbA1c: glycosylated hemoglobin

HbA1C	MILD		MODERATE	SEVERE	TOTAL
<7	3		2	1	6
7-8	7		10	4	21
8-10	9		11	7	27
>10	2		5	4	11
TOTAL	21		28	16	65
INCIDENCE OF DIABETIC COMPLICATIONS AMONG COVID-19 PATIENTS					
COMPLICATION		NO. OF PATIENTS		PERCENTAGE (%)	P-VALUE
DIABETIC KETOACIDOSIS		10		15.4	0.0001
HHS		4		6.1	0.684
SGLT2 INHIBITOR USE		11		16.9	0.0001
HYPERGLYCEMIA (>250 MG/DL)		22		33.8	0.0001

Among the 21 patients with mild disease, three had HbA1c less than 7, seven patients had HbA1c between 7 and 8, nine between 8 and 10, and two above 10. Out of the 28 patients with moderate disease, two had HbA1c less than 7, 10 had HbA1c between 7 and 8, 11 between 8 and 10, and 5 above 10. Out of the 16 patients with severe disease, one had HbA1c of less than 7, four between 7 and 8, seven between 8 and10, and four above 10. P-value was calculated using the chi-square test and was found to be significant (0.005).

In our study, 10 patients had DKA, four patients had HHS, and 22 patients had hyperglycemia (>250 mg/dl) at the time of admission. Additionally, 11 patients were found to be using SGLT2 inhibitors. It is evident from our study that the incidence of complications like DKA, HHS, and hyperglycemia in patients with COVID-19 is high; mortality and morbidity were also high among patients presenting these complications and higher HbA1c levels. This implies a stronger association between diabetes and COVID.

Oxygen therapy

The need for more oxygen therapy and ventilatory support was observed in patients with HbA1c above 8, and it was found to be statistically significant, as shown in Table 4.

**Table 4:** Duration of Oxygen Therapy Across Various Groups of HbA1c Levels and Disease Severity HbA1c: glycosylated hemoglobin

HbA1c	MILD	MODERATE	SEVERE	P-VALUE
<7	1	5	1	<0.001
7–8.5	3	11	3	
>8.5	1	6	8	

Duration of hospital stay and HbA1c

The mean hospital stay of patients in the mild group was 6.12 ± 4.36 days, in the moderate group, it was 8.69 ± 4.65, and in the severe group, it was 13.21 ± 5.67; this was found to be statistically significant. The mean ICU stay of patients in the mild group was 7.85 ± 3.21 days, in the moderate group, it was 10.45 ± 3.56, and in the severe group, it was 11.56 ± 6.79; this was found to be statistically significant. The average ICU stay and hospital stay were greater in patients with HbA1c above 8.

Morbidity and mortality index

Almost 80% of patients with HbA1c above 8 had moderate to severe disease, along with other elevated inflammatory markers. Out of the 65 patients, 12 patients succumbed to the disease. The mortality rate of the study was found to be 18.5%. Mortality was higher in patients with HbA1c above 8. Out of 46 patients, 10 patients (21.7%) had succumbed to the disease. Of patients with HbA1c levels less than 8, two (10.5%) had succumbed to the disease.

## Discussion

In this study involving acute COVID pneumonia, HbA1c was not considered a diagnostic standard in diabetic patients, as per the guidelines [10]. A lot of studies have reported that in diabetic patients contracting COVID-19 infection, there was evidence of more severe inflammation and a higher mortality rate when compared to nondiabetic patients contracting COVID-19 pneumonia [11]. Several studies showed that among patients with diabetes mellitus, the risk of severe disease and guarded outcomes are significantly high [12-14].

In patients with long-standing uncontrolled diabetes, more target organ damage is inevitable; this may lead to severe inflammatory response, hypercoagulable state, low oxygenation saturation, and higher mortality index among those with COVID-19 infection [14-19]. It was evident from our study that in patients with higher levels of HbA1c, inflammatory markers and coagulation factors were significantly elevated. In our study, similar to various other studies, various markers like CRP, ESR, and coagulation factors like D-dimer and CT-severity score correlated positively with the HbA1c level of the COVID-19 patients [15].

In our study, similar to studies by Ahmed et al., Francone M et al., and Xiong et al., the majority of the patients were middle-aged men, which was statistically significant [20]. We noticed in our study that patients with longstanding uncontrolled diabetes developed a severe form of the disease, particularly elderly men, whose CT severity score and inflammatory markers were higher than persons with no comorbid conditions, and their disease outcomes were poorer [21-23].

Observations from our study, similar to the French multicenter study CORONADO and Feldman et al., suggest that people with prolonged uncontrolled diabetes mellitus have more morbidity in the form of increased duration of hospitalization and need for a prolonged duration of ventilator support and oxygen therapy [24-26].

Our findings, in accordance with Homan et al., showed increased severity of the disease among patients with type 1 diabetes and increased incidence of complications like diabetic ketoacidosis, hyperosmolar hyperglycemic state, and euglycemic ketoacidosis [27-29]. These complications also showed a high incidence of mortality [29].

Similar to Ray et al., our study findings also showed patients with uncontrolled diabetes mellitus and other co-morbidities at an increased risk of prolonged hospitalization and oxygen therapy when infected with COVID-19. Elderly male patients with multiple co-morbidities show an especially high risk of mortality following COVID infection [30]. One could therefore expect high levels of HbA1C to predict a more severe course in patients with COVID-19. Nonetheless, the results from our study are consistent with the findings of several recent studies.

## Conclusions

Diabetes is one of the major risk factors for COVID-19, especially in the elderly population, as it considerably increases their mortality. Disease severity, duration of hospitalization and ICU stay, and the need for oxygen therapy and ventilatory support were greater in patients with higher HbA1c values, along with elevation in other inflammatory markers. The incidence of mortality was greater in patients with prolonged uncontrolled diabetes mellitus or with higher levels of HbA1c. Mortality is especially elevated in patients presenting diabetes-related complications, such as DKA, euglycemic ketoacidosis, and HHS; the mortality incidence was relatively greater.

## References

[1] Fadini GP, Morieri ML, Longato E, Avogaro A (2020). Prevalence and impact of diabetes among people infected with SARS-CoV-2. J Endocrinol Invest.

[2] Yang J, Zheng Y, Gou X (2020). Prevalence of comorbidities and its effects in patients infected with SARS-CoV-2: a systematic review and meta-analysis. Int J Infect Dis.

[3] Zhou F, Yu T, Du R (2020). Clinical course and risk factors for mortality of adult inpatients with COVID-19 in Wuhan, China: a retrospective cohort study. Lancet.

[4] Du Y, Tu L, Zhu P (2020). Clinical features of 85 fatal cases of COVID-19 from Wuhan. A retrospective observational study. Am J Respir Crit Care Med.

[5] Guan WJ, Liang WH, Zhao Y (2020). Comorbidity and its impact on 1590 patients with COVID-19 in China: a nationwide analysis. Eur Respir J.

[6] Li B, Yang J, Zhao F (2020). Prevalence and impact of cardiovascular metabolic diseases on COVID-19 in China. Clin Res Cardiol.

[7] Grasselli G, Zangrillo A, Zanella A (2020). Baseline characteristics and outcomes of 1591 patients infected with SARS-CoV-2 admitted to ICUs of the Lombardy Region, Italy. JAMA.

[8] Richardson S, Hirsch JS, Narasimhan M (2020). Presenting characteristics, comorbidities, and outcomes among 5700 patients hospitalized with COVID-19 in the New York City area. JAMA.

[9] Lighter J, Phillips M, Hochman S, Sterling S, Johnson D, Francois F, Stachel A (2020). Obesity in patients younger than 60 years is a risk factor for COVID-19 hospital admission. Clin Infect Dis.

[10] Good CB, Kolb NR, Meyer M (2019). World Health Organization guidelines on medicines for diabetes treatment intensification. Ann Intern Med.

[11] The Novel Coronavirus Pneumonia Emergency Response Epidemiology Team (2020). The epidemiological characteristics of an outbreak of 2019 novel coronavirus diseases (COVID-19) - China, 2020. China CDC Wkly.

[12] Bode B, Garrett V, Messler J, McFarland R, Crowe J, Booth R, Klonoff DC (2020). Glycemic characteristics and clinical outcomes of COVID-19 patients hospitalized in the United States. J Diabetes Sci Technol.

[13] Liu SP, Zhang Q, Wang W (2020). Hyperglycemia is a strong predictor of poor prognosis in COVID-19. Diabetes Res Clin Pract.

[14] Wang X, Liu Z, Li J (2020). Impacts of type 2 diabetes on disease severity, therapeutic effect, and mortality of patients with COVID-19. J Clin Endocrinol Metab.

[15] Guo W, Li M, Dong Y (2020). Diabetes is a risk factor for the progression and prognosis of COVID-19. Diabetes Metab Res Rev.

[16] Kornum JB, Thomsen RW, Riis A, Lervang HH, Schønheyder HC, Sørensen HT (2008). Diabetes, glycemic control, and risk of hospitalization with pneumonia: a population-based case-control study. Diabetes Care.

[17] Martins M, Boavida JM, Raposo JF (2016). Diabetes hinders community-acquired pneumonia outcomes in hospitalized patients. BMJ Open Diabetes Res Care.

[18] Levin AT, Hanage WP, Owusu-Boaitey N, Cochran KB, Walsh SP, Meyerowitz-Katz G (2020). Assessing the age specificity of infection fatality rates for COVID-19: systematic review, meta-analysis, and public policy implications. Eur J Epidemiol.

[19] Dartmouth College. (2021, January 21 (2022). Dartmouth College. COVID-19 is dangerous for middle-aged adults, not just the elderly: Study examines infection fatality rates for COVID-19. https://www.eurekalert.org/news-releases/625275.

[20] Ahmed MZ, Ahmed O, Aibao Z, Hanbin S, Siyu L, Ahmad A (2020). Epidemic of COVID-19 in China and associated psychological problems. Asian J Psychiatr.

[21] Fernández RS, Crivelli L, Guimet NM, Allegri RF, Pedreira ME (2020). Psychological distress associated with COVID-19 quarantine: latent profile analysis, outcome prediction and mediation analysis. J Affect Disord.

[22] Ghosh B, Kumar N, Singh N, Sadhu AK, Ghosh N, Mitra P, Chatterjee J (2020). A quantitative lung computed tomography image feature for multi-center severity assessment of COVID-19 [Preprint]. medRxiv.

[23] Dalal J, Triulzi I, James A (2021). COVID-19 mortality in women and men in sub-Saharan Africa: a cross-sectional study. BMJ Glob Health.

[24] CDC COVID-19 Response Team (2020). Preliminary estimates of the prevalence of selected underlying health conditions among patients with coronavirus disease 2019 - United States, February 12-March 28, 2020. MMWR Morb Mortal Wkly Rep.

[25] Feldman EL, Savelieff MG, Hayek SS, Pennathur S, Kretzler M, Pop-Busui R (2020). COVID-19 and diabetes: a collision and collusion of two diseases. Diabetes.

[26] Cariou B, Hadjadj S, Wargny M (2020). Phenotypic characteristics and prognosis of inpatients with COVID-19 and diabetes: the CORONADO study. Diabetologia.

[27] Holman N, Knighton P, Kar P (2020). Risk factors for COVID-19-related mortality in people with type 1 and type 2 diabetes in England: a population-based cohort study. Lancet Diabetes Endocrinol.

[28] Li X, Xu S, Yu M (2020). Risk factors for severity and mortality in adult COVID-19 inpatients in Wuhan. J Allergy Clin Immunol.

[29] Nagano K, Kamimura T, Kawai G (2022). Interaction between a fluoroquinolone derivative and RNAs with a single bulge. J Biochem.

[30] Ray A, Chaudhry R, Rai S, Mitra S, Pradhan S, Sunder A, Nag DS (2021). Prolonged oxygen therapy post COVID-19 infection: factors leading to the risk of poor outcome. Cureus.

